# Supplementary figures and images for: Low iron-induced small RNA BrrF regulates central metabolism and oxidative stress responses in Burkholderia cenocepacia
Source: PLoS One. 2020 Jul 23;15(7):e0236405. doi: 10.1371/journal.pone.0236405 (PMC7377471; doi:10.1371/journal.pone.0236405)

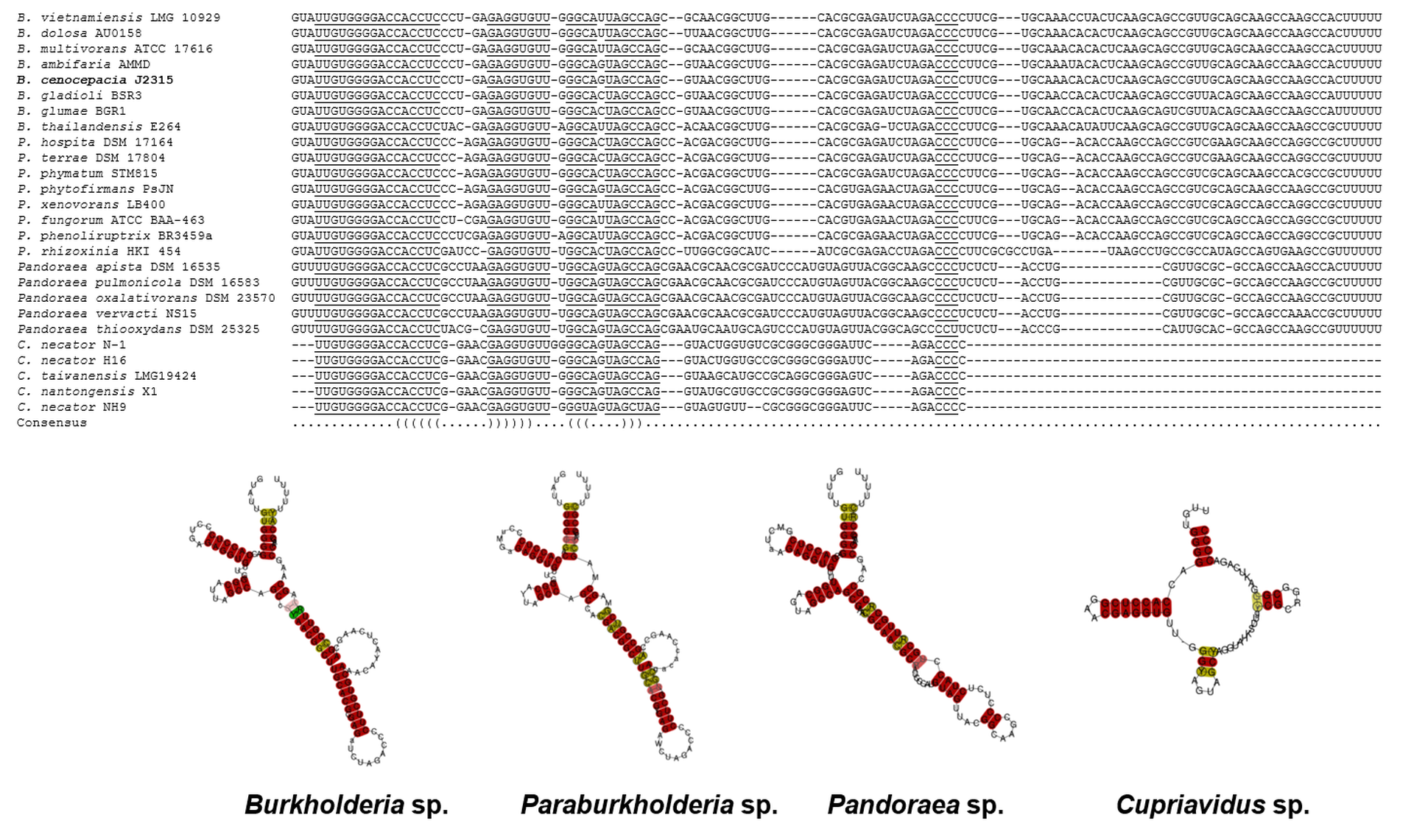

Supplement: S1 Fig — The full length of BrrF is very conserved throughout Burkholderia (first 8 lines), Paraburkholderia (lines 9–16) and Pandoraea sp. (lines 17–21). In Cupriavidus sp. (lines 22–26) only the first 45 nt of BrrF are present. Underlined are bases conserved in all sequences. Consensus secondary structures were computed using the sequences of the alignment. Red: fully conserved compatible base pairs. Alignment and consensus structures were computed using LocARNA [25]. (TIF) [file pone.0236405.s001.tif]

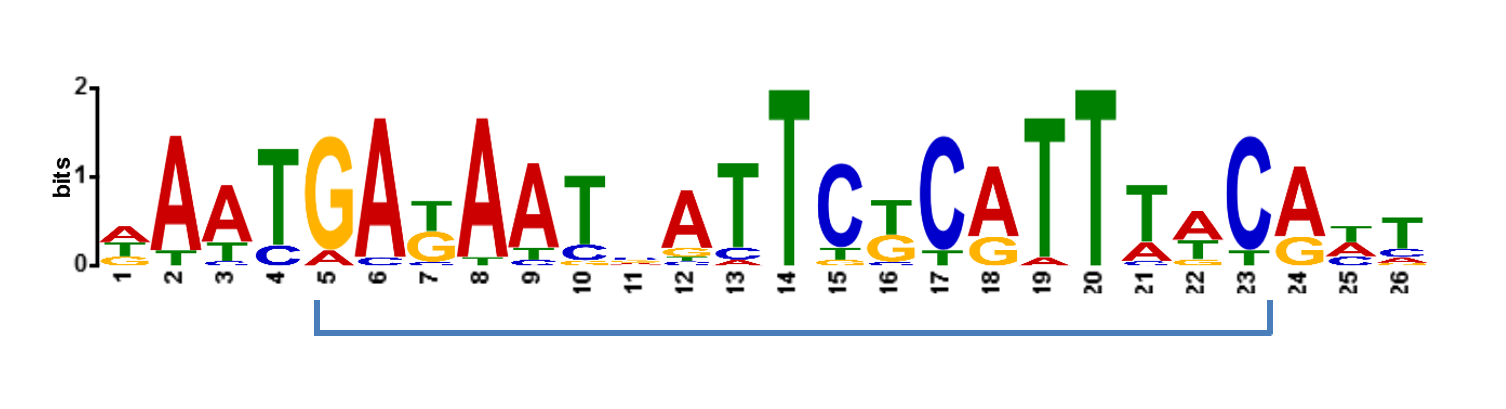

Supplement: S2 Fig — The canonical 19 bp palindromic Fur binding site is indicated by a bracket. (TIF) [file pone.0236405.s002.tif]

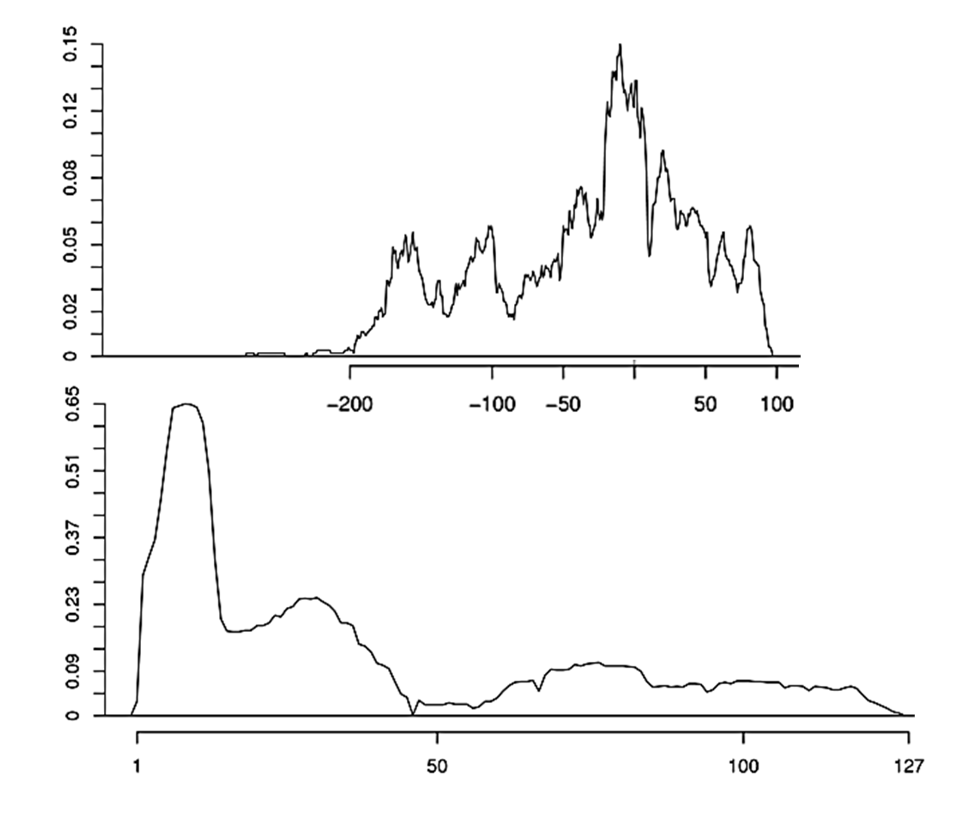

Supplement: S3 Fig — mRNA regions are depicted in the upper panel and sRNAs regions in the lower panel. The graphs represent all predictions with P< 0.01. x-axis depicts the nucleotide position, position 1 in mRNA is the first nucleotide of the coding sequence. The y-axis depicts the relative frequency of a nucleotide position being part of the predicted sRNA–target interactions. (TIF) [file pone.0236405.s003.tif]

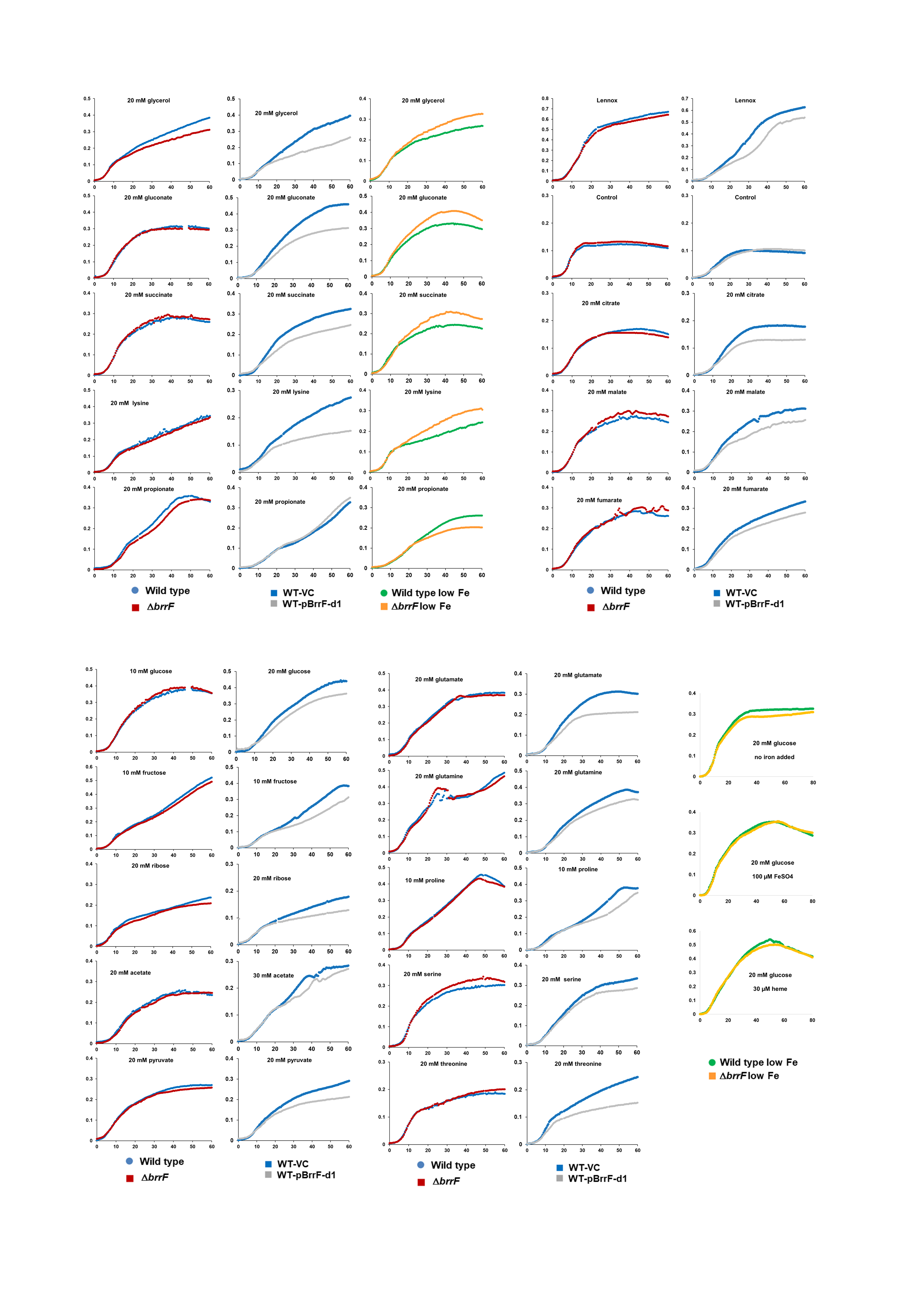

Supplement: S4 Fig — Growth was monitored in microtiter plates. y-axis: Optical density (590 nm). x-axis: Time (hours). (TIF) [file pone.0236405.s004.tif]
