# Supplementary material for: Low iron-induced small RNA BrrF regulates central metabolism and oxidative stress responses in Burkholderia cenocepacia
Source: PLoS One. 2020 Jul 23;15(7):e0236405. doi: 10.1371/journal.pone.0236405 (PMC7377471; doi:10.1371/journal.pone.0236405)
Supplement: S1 Table — (PDF) [file pone.0236405.s005.pdf]

**Table S1:** Strains and plasmids used in this study.

| | | |
|---|---|---|
| **Strains** | | |
| *B. cenocepacia* J2315 | Wild type (Wt), ET-12 lineage, CF isolate, LMG 16656 | Lab collection |
| Δ*brrF* | J2315 *brrF* deletion mutant | This study |
| Wt-VC | J2315 + pSCrhaM2 | [8] |
| Wt-pBrrF-d1 | J2315 + pM2-*brrF*-d1 | This study |
| Wt-pBrrF-d2 | J2315 + pM2-*brrF*-d2 | This study |
| Wt-pBrrF-d3 | J2315 + pM2-*brrF*-d3 | This study |
| Δ*brrF*-VC | Δ*brrF* + pSCrhaM2 | This study |
| Δ*brrF*-pBrrF-d1 | Δ*brrF* + pBrrF-d1 | This study |
| Δ*brrF*-pBrrF-d2 | Δ*brrF* + pBrrF-d2 | This study |
| Δ*brrF*-pBrrF-d3 | Δ*brrF* + pBrrF-d3 | This study |
| **Plasmids** | | |
| pSCrhaM2 | Derivative of pSCrhaB2, RBS and start codons removed, $ori_{pBBR1}$, *rhaR* and *RhaS*-$P_{rhaB}$, $Tp^r$ | [8] |
| pBrrF-d1 | pSCrhaM2 overexpressing sRNA BrrF from 80 nt upstream of the putative processing site to 49 nt downstream of the computationally predicted terminator | This study |
| pBrrF-d2 | Derivative of pBrrF-d1, overexpressing BrrF from the putative processing site to 49 nt downstream of the computationally predicted terminator | This study |
| pBrrF-d3 | Derivative of pBrrF-d1, with point mutations at position 8 and 9, replacing guanosine residues with cytosine residues | This study |
