# Supplementary material for: Low iron-induced small RNA BrrF regulates central metabolism and oxidative stress responses in Burkholderia cenocepacia
Source: PLoS One. 2020 Jul 23;15(7):e0236405. doi: 10.1371/journal.pone.0236405 (PMC7377471; doi:10.1371/journal.pone.0236405)
Supplement: S2 Table — (PDF) [file pone.0236405.s006.pdf]

Table S2: Primers used in this study.

Primers	Oligonucleotide sequence, 5′–3′	
**Insert for BrrF overexpression mutant OV1**		
S63ov-F-SpeI	AAAACTAGTCAGCATCGCGCATAACGGAGAGAC	
S63ov-R-Hind	TTTAAGCTTACATACACGCGGGCCGCATG	
**Shorter insert, from cleavage site of *brrF*, by inverse PCR (pBrrF-d2)**		
pSC-TSS-BrrF-d2-F	GTATTGTGGGGACCACCTCCCTG	
pSC-TSS-R2	ATTACGACCAGTCTAAAAAGCGCCTG	
**Point mutation of *brrF*, by inverse PCR (pBrrF-d3)**		
pSC-TSS-BrrF-d3-F	GTATTGTGCCGACCACCTCCCTGAGAG	
pSC-TSS-R2	ATTACGACCAGTCTAAAAAGCGCCTG	
**Amplification of plasmid inserts for Sanger sequencing**		
rha1F	CACGTTCATCTTTCCCTGGT	
rha1R	CTCTCATCCGCCAAAACAGC	
**Constructing and confirming deletion mutant Δ*brrF***		
preS63F-Nhe	AAAGCTAGCGACGCGCACACCGATCCTCTAC	amplification of upstream flanking region
preS63R-Bgl	AAAAGATCTGGAGGTGGTCCCCACAATACTCG	
postS63F-Bgl	TTTAGATCTCCAAGCCACTTTTTTGGTTCTCG	amplification of upstream flanking region
postS63R-Sma	AAACCCGGGGCGTGTGGTGCAAGTACCTGTG	
joinS63F	TCAAACCGAATGACCGACAC	amplification across *brrF* locus to confirm deletion
joinS63R	GGGATTCTGAAGCGACTTGC	
**qPCR primers**		
M0918F	GAGATGAGCACCGATCACAC	RNA polymerase *rpoD*, control gene
M0918R	CCTTCGAGGAACGACTTCAG	
S63F	AGAGGTGTTGGGCAGTAGCC	*brrF,* anneals at nt 24 - 43
S63R	GGCTGCTTGAGTGTGTTTGC	*brrF,* anneals at nt 82 - 101
M0967F	TCGTCAAGCTGATCGTCCTC	*sdhC*
M0967R	CGATCGAGACGACAAAGACG	

M0969F<br>M0969R	CAACGGTTTCTACGCAGTGG<br>TGGTTCTTCACGTGCTCGAC	*sdhA*
M0961F<br>M0961R	CGTTCGACACGTTCAAGGTC<br>ATCATCGTCGTGTGGCTGTC	*acnA*
M2701F<br>M2701R	ACAACATCACGACCGACCAC<br>TCGCGTACGAATTGAAGTCC	*acnM*
L3299F<br>L3299R	AATCGATGGGCTTCAAGACG<br>GACCTTCCGGGTTCACGTAG	*katB*
M0931F<br>M0931R	CGAAGTGGGACCTGTACGTG<br>CAGACCTTCGTGTCGTCGAG	catalase
L2757F<br>L2757R	ACATCGTGTCGACGAGCAAC<br>CGAAGTCCCAGTTCACGATG	*sodB*
L2143F<br>L2143R	GGCTGCATCACTTCTTCACG<br>GCCAGTTGAACACCTTCACG	*cyoB*
L2343F<br>L2343R	AGTCGGACGTGATGATCGTG<br>CGGACCACCGAGTACGAGTAG	*nuoB*
L2908F<br>L2908R	GATCGACCTGTTGCTGAACG<br>TCAGTGTCGTGCCTTCCTTG	*fumC*
L2287F<br>L2287R	GCTACTACCACCCGCTCGAC<br>CGTTGATCATGTCGGTGAGG	*fumA*
L3367F<br>L3367R	CATCCTGATCGACTGGAACG<br>GCTTCGGCTCCTTCGTGTAG	*edd*
L2643F<br>L2643R	CGGCTGTACCTCCTTTTCCTC<br>CAGGCCGACCAGGTTGTAG	*sodC*
M1833F<br>M1833R	GAAGTGTTCATCGGCTCGTG<br>CACAGCTTGACCGGAATGTC	*acnB*
M2107F<br>M2107R	TCTCGCGTCATTTCATGGAC<br>GGATCCTGCCAGATCAGCTC	*katA*
M0965F<br>M0965R	CGCTTACTCCCTGCTGTTCC<br>AATGCAACCTTCGGATCGTC	*mdh*
L2735F<br>L2735R	CAAGAACTACGCACGCAAGC<br>GATGCGTCGATCACTTCACC	*idh*
M0972F<br>M0972R	GCTACCCGATCGACAACCTC<br>ACCATCGTGTGCTTCGTGAC	*gltA*
