# Supplementary material for: Low iron-induced small RNA BrrF regulates central metabolism and oxidative stress responses in Burkholderia cenocepacia
Source: PLoS One. 2020 Jul 23;15(7):e0236405. doi: 10.1371/journal.pone.0236405 (PMC7377471; doi:10.1371/journal.pone.0236405)
Supplement: S4 Table — (PDF) [file pone.0236405.s008.pdf]

Table S4: All log2-fold changes in gene expression determined by qPCR.

		BrrF overexpression in iron-replete condition		Iron depletion		BrrF overexpression under iron depletion		
Background strain		WT	Δ*brrF*	WT	Δ*brrF*	Δ*brrF*	Δ*brrF*	Δ*brrF*
Gene	Locus tag	WT-pBrrF-d1 / WT-VC	Δ*brrF*-pBrrF-d1 / Δ*brrF*-VC	200 µM dipyridyl / no dipyridyl		Δ*brrF*-pBrrF-d1 / Δ*brrF*-VC	Δ*brrF*-pBrrF-d2 / Δ*brrF*-VC	Δ*brrF*-pBrrF-d3 / Δ*brrF*-VC
sdhC	BCAM0967	-0.36 (0.093)	-0.40 (0.059)	**-1.28** (0)	-0.29 (0.241)	**-1.41** (0.023)	**-1.62** (0.011)	**-1.34** (0.03)
sdhA	BCAM0969	-0.13 (0.597)	-0.10 (0.766)	**-2.25** (0)	0.24 (0.479)	**-2.28** (0.008)	**-2.48** (0.005)	**-2.24** (0.009)
acnA	BCAM0961	**-0.76** (0.028)	-0.30 (0.522)	**-4.22** (0)	-0.52 (0.401)	**-3.53** (0)	**-3.19** (0)	-0.47 (0.178)
acnM	BCAM2701	-0.46 (0.189)	-0.17 (0.829)	**-2.00** (0)	0.42 (0.568)	1.05 (0.812)	2.22 (0.309)	2.28 (0.289)
fumC	BCAL2908	**1.13** (0.003)	**1.00** (0.007)	**1.76** (0)	**0.42** (0.006)	**1.61** (0.036)	0.96 (0.248)	-0.69 (0.498)
fumA	BCAL2287	**-1.39** (0.020)	-0.69 (0.301)	**-5.38** (0)	**-1.09** (0)	**-5.11** (0)	**-3.59** (0)	-0.76 (0.08)
sodB	BCAL2757	**-1.22** (0.010)	-0.64 (0.174)	**-2.34** (0)	**0.75** (0.001)	**-4.31** (0)	**-3.49** (0)	**-1.32** (0.006)
katB	BCAL3299	-0.23 (0.864)	0.01 (1.0)	**-1.72** (0)	**0.98** (0.001)	**-2.01** (0.039)	-1.88 (0.053)	-0.54 (0.803)
catalase	BCAM0931	-0.25 (0.519)	0.04 (0.995)	**-0.83** (0)	**-0.18** (0.032)	-0.93 (0.072)	-0.82 (0.120)	-0.33 (0.73)
nuoB	BCAL2343	0.11 (0.875)	-0.07 (0.968)	**-1.20** (0)	-0.07 (0.951)	-0.58 (0.364)	-0.68 (0.252)	-0.58 (0.369)
edd	BCAL3367	-0.32 (0.740)	0.23 (0.885)	**-3.60** (0)	**-1.54** (0.001)	**-2.06** (0.001)	**-1.92** (0.002)	**-1.67** (0.005)
cyoB	BCAL2143	0.03 (0.602)	n.d.	-0.52 (0.372)	-0.19 (0.916)	n.d.	n.d.	n.d.
sodC	BCAL2643	0.04 (0.586)	n.d.	0.21 (0.300)	**0.74** (0.001)	n.d.	n.d.	n.d.
icd	BCAL2735	0.04 (0.682)	n.d.	-0.42 (0.078)	-0.00 (1.000)	n.d.	n.d.	n.d.
mdh	BCAM0965	0.02 (0.837)	n.d.	-0.15 (0.489)	**-0.41** (0.017)	n.d.	n.d.	n.d.
gltA	BCAM0971	-0.12 (0.198)	n.d.	**-0.75** (0)	-0.25 (0.134)	n.d.	n.d.	n.d.
acnB	BCAM1833	-0.17 (0.218)	n.d.	0.03 (0.993)	**0.44** (0.026)	n.d.	n.d.	n.d.
katA	BCAM2107	-0.02 (0.637)	n.d.	-0.57 (0.156)	0.15 (0.921)	n.d.	n.d.	n.d.

In brackets: p-values after Tukey’s post-hoc test of ANOVA, 0 denotes p-value < 0.001, fold changes with p ≤ 0.05 are in bold, n.d. not determined.
