# Supplementary material for: Low iron-induced small RNA BrrF regulates central metabolism and oxidative stress responses in Burkholderia cenocepacia
Source: PLoS One. 2020 Jul 23;15(7):e0236405. doi: 10.1371/journal.pone.0236405 (PMC7377471; doi:10.1371/journal.pone.0236405)
Supplement: Attachment — Submitted filename: response to Reviewers second revision.docx [file pone.0236405.s011.docx]

**Reviewer #1:** Almost all comments have been addressed adequately. I have only one remaining issue. In the abstract the authors state that BrrF is a "Fur-regulated small RNA" (line 27), but as indicated by the other reviewer in the first review Fur-regulation of BrrF is not directly determined for *B. ceenocepacia* BrrF. This is a reasonable assumption as all data are consistent with this model, but the language in the abstract needs to be modified to remove the certainty.

**Response:**

We have changed the wording of the abstract to “BrrF is a small RNA highly upregulated in *Burkholderia cenocepacia* under conditions of iron depletion and with a genome context consistent with Fur regulation.” (lines 27-28). We also softened the related statement in lines 307-8 in the main manuscript by changing the wording.

**Reviewer #2:** The authors have adequately addressed the comments from the reviewers, and overall, the work is sound.

However, I would like to encourage the authors to seriously assess their use of qRT-PCR for sRNA levels. I am still not entirely convinced that the qRT-PCR data is accurately depicting the levels of BrrF under different conditions and in different strains. The rebuttal from the authors includes the comment that "our no-RT controls showed that primers for BrrF always give signals in this order of magnitude." Does this not concern you that the primers you are using are not efficient and/or not reliable? I really am trying to be helpful, as I just want to make sure that the data are as accurate as possible. I would suggest that the authors consider employing northern blot analyses as they continue to work in the area of sRNAs. This will only serve to complement your qRT-PCR results, and it will significantly enhance your ability to analyze smaller differences in sRNA levels.

**Response:**

We performed Northern blotting for a previous publication (DOI:10.1038/s41598-017-15818-3), using DIG labelled probes and eventually exposing our hybridised membranes to X-ray film. Our lab does not have the setup nor permission for working with radioactively labelled probes. Accurate quantification of the expression levels of BrrF is to our knowledge not possible with that method, because black lines on exposed X-ray film would have to be analysed (please refer to the supplementary material of the publication https://static-content.springer.com/esm/art%3A10.1038%2Fs41598-017-15818-3/MediaObjects/41598_2017_15818_MOESM1_ESM.pdf). Northern blotting with this method will probaby be no improvement on qPCR. On the other hand, qPCR, although not perfect, is to our knowledge sufficient to show overexpression of sRNA.

The exact level of overexpression of BrrF from the vectors is dependent on the rhamnose concentration in the medium, and is by definition very similar for all constructs. Exactly replicating the BrrF expression level of wild type cells under iron depletion is not necessary for the purpose of showing that complementation does happen. The purpose of the qPCR experiments in this manuscript is monitoring the changes in levels of putative target mRNAs, and prove that those levels are declining upon BrrF overexpression. The exact fold changes were not subject of further interpretation.

We therefore feel that the additional work and cost involved in setting up Northern blotting experiments is not justified.
