# Supplementary material for: Low iron-induced small RNA BrrF regulates central metabolism and oxidative stress responses in Burkholderia cenocepacia
Source: PLoS One. 2020 Jul 23;15(7):e0236405. doi: 10.1371/journal.pone.0236405 (PMC7377471; doi:10.1371/journal.pone.0236405)
Supplement: Attachment — Submitted filename: Reviewer comments PLoS One BrrF rebuttal.docx [file pone.0236405.s010.docx]

**Manuscript “ Low iron-induced small RNA BrrF regulates central metabolism and oxidative stress responses in *Burkholderia cenocepacia*” by Sass and Coenye**

**Response to reviewers’ questions.**

**Reviewer 1:**

-Line 341-342: BfrB in *P. aeruginosa* is not regulated by PrrF (see Figure 5 in Wilderman, et al, PNAS 2004). In *E. coli*, RyhB was suggested to regulate FtnA, not BfrB, but latter studies showed that iron induction of FtnA is similarly independent of RyhB (Nandal, et al, Mol Micro 2010).

Answer:

The reviewer is correct, this was an oversight on our side and we deleted the sentence from the discussion.

-The conclusion section should be expanded to discuss the following points:

1. Figure 3. It seems counter that loss of a gene that is so highly conserved would result in a growth enhancement, especially when the loss of ryhB, PrrF, and other low-iron induced sRNAs results in a growth defect in low iron condition due to loss of iron sparing. This should at the very least be discussed in the concluding section.

Answer:

The *B. cenocepacia* strain used in this study, J2315, is a slow growing small colony variant. Strain J2315 permanently displays a growth deficiency compared to other *B. cenocepacia* laboratory strains such as K56-2 and H111. It is therefore probably less affected by iron starvation and can maintain growth for longer under those conditions compared to “normally” growing bacteria. Absence of downregulation of key metabolic genes, as in Δ*brrF*, can then increase growth, and not be detrimental to the survival of the bacteria.

We have added this rationale to the manuscript main text (lines 370-375: “This is in contrast to observations with a PrrF double deletion mutant in *P. aeruginosa*, which showed a growth defect compared to wild type under iron depletion [39]. *B. cenocepacia* J2315 is a slow growing small colony variant and possibly less affected by iron starvation. That Δ*brrF* is less growth attenuated than WT under iron limitation suggests that key metabolic genes are down-regulated by BrrF.”).

2. The authors state that many of the complementarities with BrrF targets do not overlap the SD or start site, which is contrast to other negatively regulated targets of trans-acting sRNAs. Have the authors looked at the structures of any of the target mRNAs to determine how binding by BrrF may affect access to the SD and/or start site?

Answer:

We have performed secondary structure analysis for the genes listed in Table 1, including the 5’UTR to max. 200 nt upstream of the gene start and to 100 nt downstream of the gene start. The putative interaction region was not involved in occluding the SD or the gene start, and repeating the analysis after virtual cleavage of the mRNA at the predicted interaction site did not reveal occlusion of SD or gene start after cleavage. Computational secondary structure analysis was therefore not informative regarding mechanism of action of BrrF.

We have added the findings from secondary structure analysis to the manuscript text (lines 330-332: “Secondary structure analysis of putative target mRNAs before and after virtual cleavage at the predicted interaction site did not indicate an occlusion of Shine-Dalgarno sequences by mRNA intramolecular structures.”).

3. Related, I’m curious if any complementarity was identified in the fumC mRNA - this distinction from how fumC is regulated by iron in other species (directly by Fur versus via the sRNA), is very interesting.

Answer:

Activation of expression by a small RNA is less common than downregulation. The most common mechanisms for activation are reduction of secondary structure in the region of the SD sequence, and occluding RNase sensitive sites.

Secondary structure analysis of the 5’UTR of *fumC* mRNA showed that it contains a hairpin formation at position ‑32 to ‑51 upstream of the gene start, which could act as a rho-independent terminator. The SD or gene start are not occluded by secondary structure formation according to this analysis. The binding site of BrrF is predicted for position ‑65 to ‑75, not overlapping the putative terminator structure. We repeated the secondary structure analysis after removing the RNA sequence upstream of and including the interaction site, and the terminator structure still formed unchanged. It is therefore not apparent from structure analysis what the exact mechanism of activation is.

We added the special characteristics of the *fumC* 5’UTR to the manuscript text (lines 431-441: “sRNAs capable of both repressing and activating gene expression have been described [44]. The most common mechanisms for activation are reduction of secondary structure in the region of the SD sequence, and occluding RNase sensitive sites [45]. BrrF is predicted to interact with fumC mRNA in its 5’UTR, 65 to 75 nt upstream of the gene start. Secondary structure analysis of the fumC 5’UTR revealed a rho-independent terminator-like hairpin structure at position 32 to 51 from gene start, suggesting transcription termination as a possible mechanism of FumC expression regulation. However, the putative interaction site does not overlap the hairpin structure, and cleaving the 5’UTR at the interaction site does not change the structure of the predicted hairpin. The mechanism of transcription activation by BrrF is therefore not apparent from secondary structure analysis.”).

**Reviewer 2:**

-In lines 27-28, the authors state that "BrrF is a Fur-regulated small RNA," however, in lines 296-297, the authors state that "BrrF is therefore probably under the control of the Fur regulator." There is not direct evidence that Fur regulates brrF (or hemP for that matter) in *B. cenocepacia*. The authors are relying solely on the presence of putative "Fur boxes" for this conclusion. While suggestion that brrF "might" be controlled by Fur is an acceptable proposition, there authors have not presented any direct evidence that brrF is actually Fur-regulated.

Answer:

In the particular *B. cenocepacia* strain used for this study, Fur is an essential gene (reference Wong *et al*., 2016, Candidate essential genes in *Burkholderia cenocepacia* J2315 identified by genome-wide TraDIS. Front. Microbiol. 7:1288. doi: 10.3389/fmicb.2016.01288). In other *Burkholderia* strains, Fur can be deleted, and it has been deleted in *B. multivorans*, also a member of the *Burkholderia cepacia* complex and relatively closely related to *B. cenocepacia*. The Fur-dependent expression of the HemP protein upstream of BrrF has been demonstrated for that bacterium (ref. 21). The sequences upstream of *hemP* are identical in *B. cenocepacia* J2315 and the *B. multivorans* strain used in that study, including the Fur box. The sequence upstream of *hemP* of *B. multivorans* has been used in a Fur titration assay in that study (ref. 21), showing that *E. coli* Fur protein can bind to this Fur box sequence. The same sequence was also bound by *B. multivorans* Fur in an EMSA experiment (ref. 21). It is therefore very likely that the *hemP* upstream sequence in *B. cenocepacia* is also bound by Fur.

We have added these observations to the manuscript text, lines 302-307 (“The sequence upstream of the *hemP* gene start in *B. cenocepacia* is identical to that upstream of *hemP* in *B. multivorans* for the first 77 nt, which includes the Fur box. This *B. multivorans* sequence was bound by *B. multivorans* Fur protein in an electrophoresis mobility shift assay [21], the respective sequence in *B. cenocepacia* is therefore most likely also bound by Fur.”)

-Regarding the deletion and over-expression of brrF, there are several important data that are missing. The authors have relied on qPCR to demonstrate levels of BrrF in all of their strains (Fig. S5), but the authors should employ another method, such as northern blot analysis. For example, Fig. S5 shows that the Cq value for BrrF in the brrF deletion strain in unstressed conditions is approximately 31-32, and the Cq for BrrF in the same strain under low Fe conditions is approximately 25. How can there be such a substantial increase in the levels of BrrF in the brrF deletion strain in different conditions? This shows the unreliable nature of qPCR for analyzing sRNAs. Moreover, given this issue, much of the data in the manuscript are difficult to interpret.

Answer:

The reason to show the qPCR raw data was to point out that the levels of BrrF in overexpression experiments under low iron conditions are comparable to the levels of BrrF in wild type under low iron conditions. In the *brrF* deletion mutant, Cq values for BrrF are between 25 and 32, which is indeed relatively high, given that BrrF should not have been detected. However, our no-RT controls showed that primers for BrrF always give signals in this order of magnitude, as Cq values for BrrF in no-RT controls ranged from 32 to 27. In contrast, no-RT control Cq values for all other genes investigated were always larger than 34. It appears that, for reasons unknown we had measurable *brrF* signals that were larger than for all the other genes investigated. However, whatever the reason for this high background signals, we are convinced they have no influence on our conclusions. After all, the Cq values for BrrF in wild type under iron replete conditions are approx. 15, while those under conditions where BrrF is induced are approx. 10-11. This difference between Cq values of 10-15 on one hand, and Cq values of 25-32 on the other hand, is large enough. Generally, no-RT and no-template control Cq values should be >5 cycles larger than sample Cq values, to contribute <3% to the signal (Nolan et al., 2006, Quantification of mRNA using real-time RT-PCR. Nat. Meth. 1:1559, doi:10.1038/nprot.2006.236), and this is clearly the case for our data . Because of this we believe our qPCR data are reliable and we decided not to quantify BrrF expression in another way.

We have supplied all qPCR raw Cq values, including no-RT controls, as supplementary material, in Table S5, and have removed Figure S5, since it depicts data which is now available in Table S5.

-Also regard the deletion strain, are hemP levels altered by deletion of brrF? If so, it is very difficult to conclude that any phenotypes observed are related only to BrrF.

Answer:

*hemP* mRNA levels were shown in Figure S5 on the right hand side, they are not altered by *brrF* deletion. We have now supplied all qPCR raw data in Table S5.

We conducted additional growth curves to assess the functionality of HemP. HemP is necessary for heme uptake in *B. multivorans* (ref. 29); and our results showed that a *brrF* deletion mutant was not impaired in heme utilization (Figure S4). It is therefore reasonable to assume that HemP expression is not altered by brrF deletion, and that the observed phenotypes of ΔbrrF are not related to hemP.

We have added the results of the additional growth tests to the main text (lines 368-369: “Δ*brrF* was also not impaired in heme utilisation, indicating that expression of HemP is not affected by *brrF* deletion (Fig. S4).”), and added representative growth curves, showing the effect of heme addition to the low iron medium, to Figure S4 in supplementary material.

-Figure 3 should be statistically analyzed. The authors state that strains are "marginally" different, but are these differences statistically significant?

Answer:

To perform the statistical analysis for all available data as consistently as possible, we analyzed the growth rates. Part of the growth curves in Figure 3 were obtained with a Cell Growth Quantifier, which runs with CGQuant software with automated growth rate calculation. The equation used by the CGQuant software to calculate time averaged growth rates was applied to the output of the microplate reader. The resulting growth rates were then statistical analyzed. The method section has been amended accordingly (lines 140-144: “Growth rates were determined using the equation µ * h^-1^ = ln (x_t2_/x_t1_)/(t_2_-t_1_), where x denotes optical density or backscatter arbitrary units, and t_1_ and t_2_ refer to a point at the beginning or end of the analysed time interval, respectively [17]. The obtained values for µ of the biological replicates were then analysed either by One-way ANOVA or a two-tailed Student’s t-test using SPSS (v. 25).”), as well as the legend to Figure 3 (lines 392-394: “Significant differences in growth rate of the wild type or wild type vector control culture and the respective test condition are indicated by asterisks (* = p < 0.05, ** = p < 0.01).“). Statistically significant different growth rates are indicated in Figure 3.

-Lines 453-460 and Figure 7. These data are extremely difficult to understand. To begin with, the authors use confusing terminology: "significant increase of inhibition." This appears to translate to increase sensitivity, but it is hard to know exactly.

Answer:

We have changed the wording of this sentence from “significant increase of inhibition” to “significant increase in size of inhibition zone” (line 493) to clarify.

-Regarding the data, WT vs. WT in low Fe shows no difference in sensitivity to oxidative stress, and there should be a >50-fold increase in BrrF in the WT-low Fe condition compared to WT based on the authors previous work (Ref. 9). Additionally, the brrF deletion strain in both conditions has similar sensitivity levels to those of the WT and WT-low Fe. However, when you over-express versions of brrF in the brrF deletion strain, these strains exhibit increased sensitivity to oxidative stress. Is there a greater than 50-fold increase in BrrF in these strains? Can over-expression of these brrF genes in the WT strain similarly increase sensitivity to oxidative stress. The authors need to carefully evaluate the results from these experiments.

Answer:

The variations of the effect of BrrF induction on H_2_O_2_ sensitivity in WT under low iron (not significant) and in ΔbrrF with BrrF expression complemented under low iron condition (significant) is probably not due to a difference in BrrF expression in these two strains as the reviewer suggested, but due to a reduction of *katB* expression under the condition of BrrF overexpression. *katB* is approx. 2-fold lower expressed in media containing rhamnose and trimethoprim, which are used for overexpression experiments. This increases the excess of BrrF in comparison to its target *katB* mRNA, and could cause an increase in *katB* down-regulation, which is also apparent in *katB* Cq values under this condition.

We have supplied all the qPCR raw data (Table S5), and added the above explanation to the manuscript text (lines 519-524: “Concomitantly, baseline expression of *katB* is relatively high compared to *katA*, with a 2-fold reduction in the media containing rhamnose and trimethoprim which were used for overexpression experiments (Table S5). This lower expression of *katB* could be the reason for the larger effect of BrrF overexpression on H_2_O_2_ sensitivity compared to induction of BrrF by low iron (Fig 7), as this would increase the excess of BrrF relative to its target *katB*.”).
